# A compressed sensing perspective of hippocampal function

**DOI:** 10.3389/fnsys.2014.00141

**Published:** 2014-08-08

**Authors:** Panagiotis C. Petrantonakis, Panayiota Poirazi

**Affiliations:** Computational Biology Lab, Institute of Molecular Biology and Biotechnology, Foundation for Research and Technology-HellasHeraklion, Greece

**Keywords:** hippocampus, entorhinal cortex, compressed sensing, sparse coding, episodic memory

## Abstract

Hippocampus is one of the most important information processing units in the brain. Input from the cortex passes through convergent axon pathways to the downstream hippocampal subregions and, after being appropriately processed, is fanned out back to the cortex. Here, we review evidence of the hypothesis that information flow and processing in the hippocampus complies with the principles of Compressed Sensing (CS). The CS theory comprises a mathematical framework that describes how and under which conditions, restricted sampling of information (data set) can lead to condensed, yet concise, forms of the initial, subsampled information entity (i.e., of the original data set). In this work, hippocampus related regions and their respective circuitry are presented as a CS-based system whose different components collaborate to realize efficient memory encoding and decoding processes. This proposition introduces a unifying mathematical framework for hippocampal function and opens new avenues for exploring coding and decoding strategies in the brain.

## Introduction

The rules that govern information flow between different hippocampal subregions represent the very quintessence of its main functionality: the formation and retrieval of new memories. However, various theoretical issues arise regarding those rules. For instance, in what ways—if any—are the firing rates of two interconnected regions *causally* related during memory formation? To what extent is a *sparse* neuronal population activity required for efficient memory encoding? What are the crucial *limitations* of inter-regional interactions for successful reconstruction of memory entities in a neural circuit? All above-stated queries incorporate concepts, such as, causality, sparsity, and constraints' definition, that introduce the need for a strict, mathematical interpretation.

Various models have been proposed regarding the conceptual relationship between hippocampal circuitry and function. However, none of these models included a strict mathematical formalization of their proposed theory (Lisman, 1999; Lisman and Otmakhova, 2001; Lisman et al., 2005; Cheng, 2013). On the other hand, a more mathematically oriented approach (Rolls, 2010), focused on the computational formalization for each hippocampal subregion independently, without any unifying framework that governs intra- or inter-regional relations. What is yet to be revealed is the potential of using a single mathematically formulated theory for the entire hippocampal formation. This would be the first step toward a holistic interpretation of hippocampal function and could provide new analytical tools for interpreting experimental data while paving the way for an omnibus, black-box-like modeling of hippocampus in brain networks. Toward this goal, we introduce the hypothesis that hippocampus-related regions, interact and function under the main principles of the well-defined theory of Compressed Sensing (CS) (Candes and Tao, 2006; Candès et al., 2006; Donoho, 2006; Baraniuk et al., 2010).

CS, a recent breakthrough attainment within the Signal Processing field, asserts a new encoding/decoding context. The CS theory builds upon the fundamental fact that many signals can be represented using only few (sparse), linearly combined, elements of a suitable basis or dictionary. Nonlinear optimization algorithms can then lead to recovery of such signals from very few measurements/samples, significantly fewer than the widely known Nyquist–Shannon sampling theorem implies (Nyquist, 2002). The CS theory is emerging as a key mathematical framework that can be of material value for multiple facets of neuroscience research, particularly for neuronal data analysis, fluorescence microscopy, gene-expression analysis, and connectomics (Ganguli and Sompolinsky, 2012; Mishchenko and Paninski, 2012; Advani et al., 2013). However, the possibility that compressed sensing processes can actually be implemented by neural tissue remains an elusive proposition.

Here, we provide evidence in support of a novel view of hippocampal function. The specific hypothesis that we evaluate is that Entorhinal Cortex (EC), Dentate Gyrus (DG), and the CA3 areas interact and function under the rules of CS (throughout this paper, for simplicity reasons, we refer to EC as part of the hippocampal formation). The conjectured mapping between CS and hippocampus aims to associate the conceptual meaning of each CS mathematical entity or principle with the functional role of each hippocampal region. This is the cope stone of our work: to reveal a plausible way, according to which, each hippocampal subregion contributes to the CS-based model of hippocampal function. Toward this goal, the main principles that govern CS are linked to the different aspects of memory encoding/decoding in the hippocampal formation.

We start by presenting the conceptual and mathematical framework of the CS theory. We then associate each one of the three aforementioned hippocampal related regions, i.e., EC, DG, and CA3, with certain aspects of the CS theoretical framework and describe the contributions of each region in the CS manifestation in the hippocampus. Moreover, a new, CS-based, memory encoding/decoding model for the hippocampus is introduced. Finally, conclusions along with future directions for hippocampal research exploiting the new theoretical model are discussed.

## Compressed sensing

The groundbreaking contribution of CS in the Signal Processing field is that it revealed the possibility of achieving a compressed encoding -and subsequently an efficient decoding- of a particular signal through a simple, linear measurement process. The latter dramatically reduces the number of measurements needed for efficient reconstruction, compared with the ones indicated by the Nyquist–Shannon sampling theorem. As a result, CS has led to some major advancements in the field of signal/image processing (Lustig et al., 2007). In the following paragraphs we describe the main theoretical aspects and formalization of CS and discuss the conditions under which it can be applied.

### CS basics

**Figure 1:**
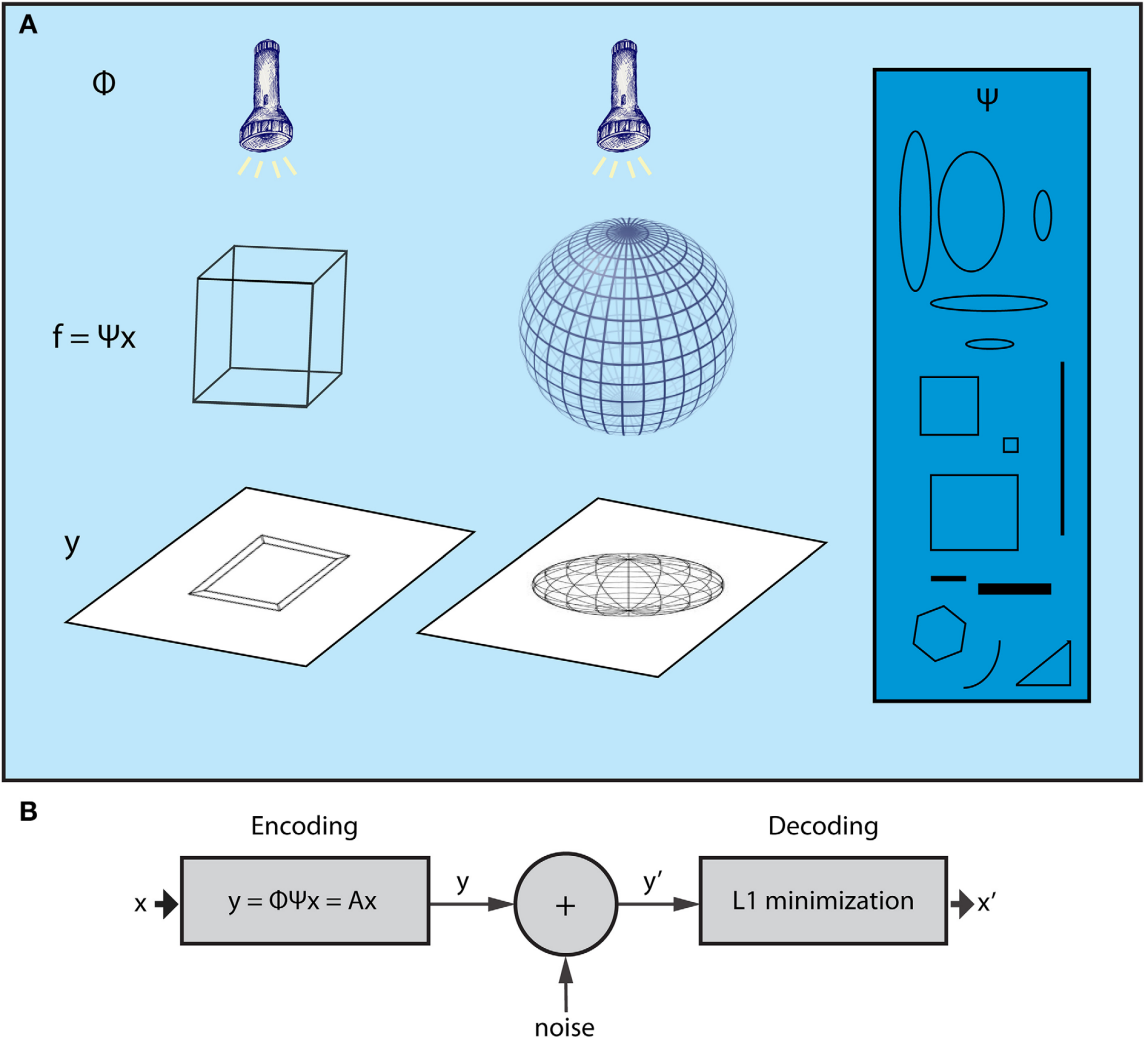
**The Compressed Sensing framework. (A)** The essence of the CS framework can be conceived if we consider the example of the wire-frame object (Ganguli and Sompolinsky, 2012). The three-dimensional object (e.g., a wire-frame cube or a wire frame sphere) is projected onto a two dimensional screen when a light beam is applied on it. The wired frame represents the *f* signal, the light beams the sampling process (Φ) whereas different shadows correspond to different samples of the signal (vector *y*). CS shows that it is possible to reconstruct the initial wire frame (e.g., the cube or the sphere) from a set of different shadows, as long as the wire frame is sparse enough and the sampling is random. For instance, consider a non-random lighting where the light beams are aligned with a specific wire of the object. The shadows would be biased to that wire and, as a result, not representative of the higher dimensional structure of the object. Moreover, in the case where the wire-frame object has dense wiring (i.e., not sparse), all shadows would be almost the same no matter what the lighting angle was. The basis Ψ (blue box) includes items that can be used to reconstruct signal *f* (the wired-object) as dictated by vector *x*, which is produced by the *L*_1_ minimization algorithm subject to measurements *y* (shadows). **(B)** CS encoding and decoding schemes. The encoding of the signal is a simple, linear sampling/measurement process derived as *y* = *Ax*, where *A* is analyzed as *A* = Φ Ψ. Thus, the decoding process is performed by knowing *A* and vector *y*′, which is a noisy version of *y*. CS theory provides mathematical proofs that, knowing *y*′ and *A*, it is possible to retrieve *x* or *x*′ ≈ *x* by a *L*_1_ minimization procedure.

The CS theory originates from the field of high-dimensional statistics. Recent advances in this field have led to a powerful, yet extremely simple methodology for dealing with the curse of dimensionality, termed Random Projections (RP). This entails a random projection of data patterns from high dimensional spaces to lower ones (Baraniuk, 2011), which reduces the dimensionality while retaining the valuable content of the original data, allowing for efficient processing in the lower dimensional space. What the CS theory adds to this framework is that, once the data with high dimensionality are represented by sparse components of a suitable basis set, it is possible to reconstruct them by their RPs! Thus, low dimensional RPs are not only suitable for interpreting the original, high dimensional data patterns but also comprise an efficient encoding that can be used as a compressed representation of the original data; high dimensional patterns can then be recovered by appropriate decoding processes. Figure 1A depicts 3D objects and their 2D shadows which can be parallelized with the high dimensional data and lower dimensional RPs, respectively. CS theory implies that it is possible to infer the form of the 3D structure using only a limited set of 2D shadows (random projections) of the wired frames.

We will now present a stricter, mathematical formalization of the CS framework. Let *f* be the *N*-dimensional signal (original data of high dimension) that we wish to measure/encode (project to a lower dimensional space). For instance, *f*, can be an *N*-dimensional vector of the gray scale intensity of pixels of a natural image. Alternatively, in the case of a spiking neural network of *N* neurons, *f* could represent the firing rate of each neuron. Now let *y* be a sampled vector from *f* with length *M* < *N*, i.e., *y* is the set of projections of signal *f* to a lower dimensional space. We can express the relationship between the signal and the sampled vector as *y* = Φ*f*, where Φ is an *M* × *N* sampling matrix (the matrix that performs the projection from one space to another). For the natural image example, *y* is a compressed version of the original image due to the projection of the *N*-dimensional space to an *M*-dimensional space via Φ. For the neural network case, *y* can be considered as the projection from one brain area to another via a convergent axonal pathway. For example, this could represent the projection from the cortex (signal *f*) to CA3 via perforant path. In this case *y* would be the activity (firing rates) of a subset of *M* neurons in the CA3 region.

**Figure 2:**
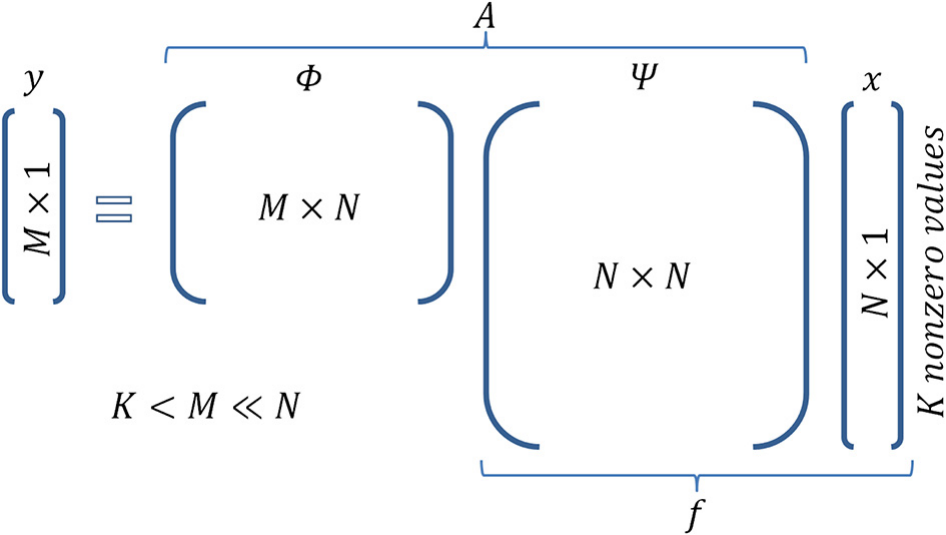
**Matrix operations in Compressed Sensing**. Figure illustrates the encoding equation of CS *y* = *Ax* and the corresponding dimensionality. It is actually a combination of the equationis *f* = Ψ *x* (sparse representation of signal *f*) and *y* = Φ *f* (sampling of signal *f*).

Furthermore, assume that the given signal can be represented by a basis set according to the equation *f* = Ψ *x*, where Ψ is a *N* × *N* matrix whose columns are the components of the basis set, and *x* is the *N* × 1 vector which contains the coefficients that analyze the signal *f* in basis Ψ. Note that *x* is sparse, i.e., it has *K* « *N* nonzero values. For instance, in the case of a gray scale natural image, Ψ could be a wavelet basis set. For the neural network example, the basis set can be represented by the activity of cells that exhibit certain properties, regarding, e.g., their receptive fields, such as mammalian visual cortex cells (Olshausen and Field, 1996), or their spatial firing patterns, such as grid cells (Hafting et al., 2005). Cells with such activity properties can form a *basis set* if their (appropriate) combination can generate any signal *f* (activity pattern of neurons) in the cortex. Figure 1A intuitively illustrates the meaning of each one of the above entities in accordance with the wire frame paradigm whereas Figure 2 graphically illustrates the mathematical formalization of the CS framework in a matrix-like operation. In the following section we elaborate more on the measurement/encoding (projection to a low dimensional space) and decoding (high dimensional space data reconstruction) phases according to the CS theory. Figure 1B graphically depicts the aforementioned processes.

### Encoding

According to the CS theory, the encoding of a given signal is a simple, linear sampling/measurement process whereby *y* = *Ax* (Figure 1B) and *A* = Φ Ψ (equations *f* = Ψ *x* and *y* = Φ *f* are combined). Matrix *A* is frequently referred as the measurement or the sensing matrix. Thus, having determined the *x* vector according to basis Ψ, we can extract a measurement/encoding vector by a mere matrix multiplication. At this point, it is obvious that algebraic multiplications, as expressed in the CS formalization, are straightforward in the natural gray scale image example. Yet, they are not valid in a spiking neural network. Rather, in that case, multiplication can be thought of as the influence of one region to another. For instance, equation *y* = *Ax* of CS formulas, can be interpreted as the concerted influence of regions whose activities are described by matrix *A* and vector *x* to the region whose activity corresponds to vector *y*.

It should be noted that in order to achieve an efficient and reversible encoding process, matrices Φ and Ψ, and subsequently matrix *A* must fulfill three very important conditions termed *sparsity*, *incoherency*, and i*sometry*. The following paragraphs elaborate on these conditions along with the role of *randomness* on their fulfillment within the CS framework.

#### Sparsity

The first condition entails that Ψ must be a sparsity basis. This condition is met if the *x* vector is sparse, i.e., has very few nonzero elements. Thus, only few components from basis Ψ are required in order to represent signal *f*. For instance, in case of a Fourier basis set, Ψ, sparsity implies that the majority of the energy of signal *f* is contained in a few frequency components. Concerning the natural gray scale image example, very few coefficients are needed to represent the image via a wavelet basis set, thus the *x* vector is sparse. Regarding the spiking neural network example, sparse representations (activity of few neurons) in one area may encode redundant activity of many neurons in upstream areas. In fact, CS, exploits the natural rule which states that many signals are sparse when they are expressed in a proper basis Ψ (Candes and Wakin, 2008). Sparsity is the crucial property in the CS framework, as without sparse representation in the higher dimensional space, the lower dimension random projections (vector *y*) are not sufficient for effective reconstruction. Note, however, that the number, *K*, of the representative components of a signal in a particular basis, i.e., the number of nonzero elements of *x*, is not always known a priori. Yet, this is not a limitation for CS, as the main constraint is the sparsity itself and not the actual representation of the signal.

#### Incoherency

Next, we must define a suitable sampling matrix Φ, given a basis Ψ. According to the CS theory, matrices Φ and Ψ must be as incoherent as possible. Incoherency implies that any component of the matrix Φ (Ψ) has dense (exactly the opposite of sparse) representation in the matrix Ψ (Φ). As a result, many components (columns) of Ψ are needed to represent each component in the measurement matrix Φ and vice versa.

To conceive the importance of the incoherency property assume a chessboard and consider each square on the chessboard as a component (column) of the basis Ψ, thus *N* = 64. Assume that there are three chess pieces (*K* = 3) on the chessboard and you are asked to find their location (A–H, 1–8) and type (e.g., white tower, black king, etc.) given only five choices (*M* = 5). If one decides to pick one square at a time, then the sampling components coincide with the basis' components and maximum coherency is accomplished. As a result, the probability of finding the three chess pieces with only five choices is extremely low. Moreover, if the number of the chess pieces, *K*, is unknown, the only way to find the position of all pieces would be to choose all squares in the chessboard, thus *M* = *N*. However, if one enlarges the sampling square by using blocks of squares for each choice, the possibility of finding the three pieces increases. In this way, each sampling square (block of squares) has a more dense representation on the Ψ basis (chessboard), as two, three, four etc., single squares are used to construct a sampling component. A set of these sampling components is used to construct the matrix Φ. The latter becomes increasingly incoherent to Ψ as sampling components (blocks of squares) become larger, increasing, at the same time the probability of finding the three chess pieces with five sampling choices.

#### Restricted Isometry Property (RIP)

**Figure 3:**
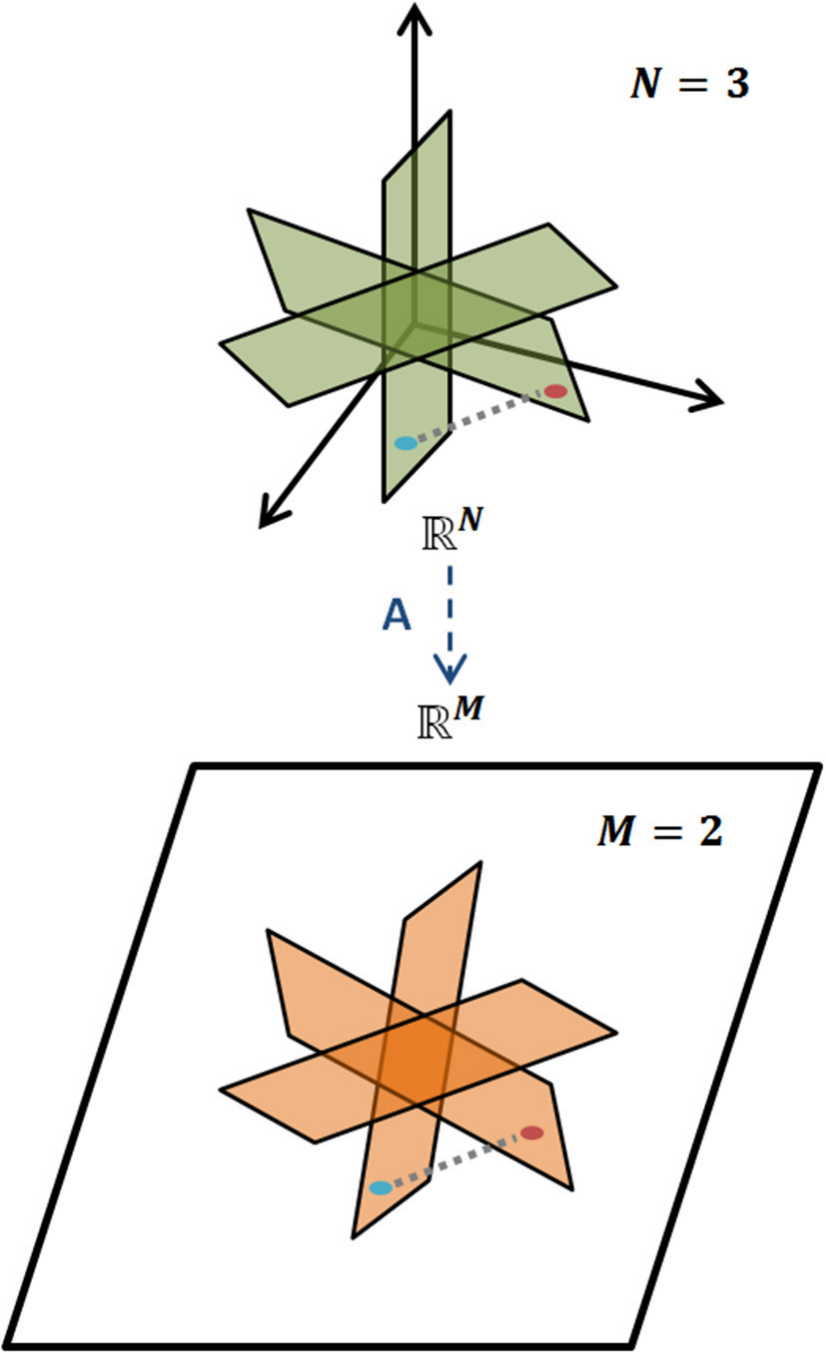
**Restricted Isometry Property**. Illustration of RIP for a *K*-sparse model of signals, where geometric information is preserved when mapped, via *A*, from the *N*-dimensional space to the *M*-dimensional one, *M* < *N*.

According to CS, the matrix *A* must obey the Restricted Isometry Property (RIP) (Candes and Tao, 2005) as a fundamental condition for efficient encoding and reconstruction/decoding. Specifically, for a predefined integer, *K*, there must be an isometry constant, δ_*K*_, of a matrix *A* such that:

(1) $$\left(\text{1}-\delta_{K}\right)\;{\left\|x\right\|}_{L_{2}}^{2}\;\leq \;{\left\|Ax\right\|}_{L_{2}}^{2}\;\leq \;\left(1\;+\;\delta_{K}\right)\;{\left\|x\right\|}_{L_{2}}^{2}$$

holds for all *K*-sparse vectors *x*, i.e., for all vectors *x* that have exactly *K* nonzero elements. The *L*_2_ norm is the magnitude of a vector. Loosely, a matrix *A* obeys the RIP of order *K* if δ_*K*_ is sufficiently smaller than one (Candes and Wakin, 2008). Intuitively, RIP entails that all pairs of vectors, *x*_*i*_, *x*_*j*_, which are *K*-sparse in Ψ, preserve their between distance even after the projection to the *M*-dimensional space through matrix *A*. This preserves the geometric properties of the vectors in the projected/measurement space and ensures an efficient decoding. A representative example of RIP is graphically illustrated in Figure 3.

#### Radomness

All the constraints introduced by CS for matrices Φ, Ψ, and *A* are fundamental for the encoding stage. Surprisingly, it can be proved that randomness constitutes the main ingredient for the construction of such matrices. Moreover, according to the CS theory, random matrices are sufficiently incoherent with any given basis matrix, Ψ, where a signal *f* has concise representation. For instance, for a fixed basis matrix Ψ, it can be proved that random waveforms used as columns for matrix Φ, with independent identically distributed (i.i.d) entries (e.g., gaussian or binary) exhibit, with high probability, very low coherence with Ψ. Furthermore, the sensing matrix *A* meets the RIP property with overwhelming probability if it incorporates i.i.d. entries from various distributions (Gaussian, Bernoulli etc.). One crucial result of CS is that with such random measurements, only:

(2) M ≥ C · K · log(N/K)

samples are needed for efficient reconstruction (*C* is an application specific constant). It can be proved analytically that randomized sampling along with the decoding process described in the subsequent section constitute a near-optimal sensing strategy (Candes and Wakin, 2008). Finally, it should be noted that randomness is a sufficient but not a necessary condition for incoherency. For instance, it is possible to find two matrices, Φ and Ψ that are incoherent but not random. In fact, it was recently shown that certain types of nonrandom matrices exhibit the same decoding performance (see Section Decoding) as random ones (Monajemi et al., 2013).

### Decoding

In many applications *y* is not the exact vector as in the encoding process as it can be affected by noise (Figure 1B), e.g., transmission noise through a communication channel. In a neuronal network paradigm, noise could be considered, for instance, as the slight difference in neuronal patterns activated during recall from the pattern that was active during the formation of a memory. Thus, the decoding process is performed given *A* and a noisy *y*′.

The set of equations determined by *y* = *Ax* do not have a unique solution as *M* < *N*. Nevertheless, the CS theory provides analytical verifications that, given *y*′ and *A*, it is possible to retrieve *x* or *x*′ ≈ *x* (and therefore *f*′ ≈ *f* due to *f* = Ψ *x*) by a nonlinear recovery/decoding process, termed the *L*_1_ minimization process. *L*_1_ minimization is based on the minimization of the *L*_1_ norm and is formulated as:

(3) $$min||x^{\prime }|{|}_{L_{1}}\text{subject to }||\;Ax^{\prime }\;-\;y^{\prime }\;{||}_{L_{2}}\leq \;\varepsilon$$

The *L*_1_ norm of a vector is simply the sum of the absolute values of its elements whereas the *L*_2_ norm is the magnitude of the vector. ε is just a bound on the noise. There are various efficient, and computationally tractable algorithms that lead to a solution of (3) (Maleki and Donoho, 2010), even for large numbers of entries in *A* and *x*.

## Measurement (Φ) and basis (Ψ) sets in the hippocampus

**Figure 4:**
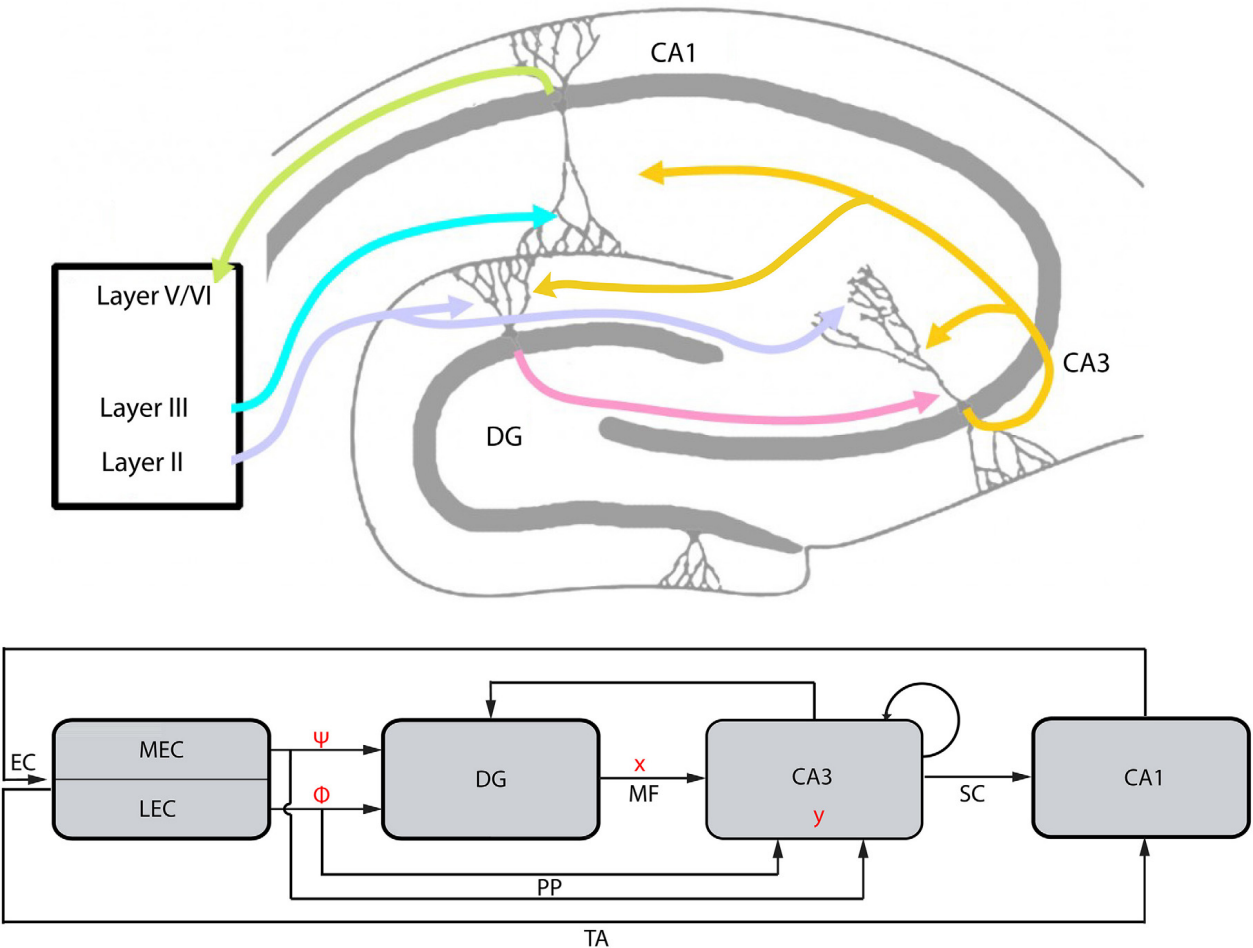
**The EC-DG-CA3-CA1 circuit and the conceptual parallelism with the CS framework (top: hippocampal structure; bottom: abstract graphical representation of hippocampal structure and conceptual parallelism with CS framework)**. The EC projection to DG and CA3, via the perforant path (PP), represents the matrix *A* of the CS framework. It is analyzed to matrices Φ and Ψ that are linked with subregions LEC and MEC, respectively. Vector *x* corresponds to the activity of DG afferents projecting to CA3 (mossy fibers, MF). Input to the CA3 from PP and MF lead to the activation of a CA3 population, the vector *y*. Backprojection from CA3 to DG performs error correction tasks in order to transform a noisy version of the vector *y*′ to *y* while recurrent collaterals within CA3 are assumed to perform association tasks. CA3 projects to CA1 via the Schaffer Collaterals (SC) and the information loop closes by the CA1 feedback to the EC, which also receives the EC input via the Temporoammonic pathway (TA).

Memory formation in the hippocampus depends on processing of information from distinct subregions. Information passes from one subregion to the other toward a more efficient encoding (Amaral, 1993). We assume that in the EC-DG-CA3 circuit (Figure 4), information to be stored/processed is transferred from EC to CA3 via the DG to achieve an orthogonal encoding, i.e., more distinguishable from other memory entities, and more information-rich (compressed) than in its origin. Having established the conceptual framework of CS, we propose that an equivalent transformation of information is performed in the hippocampus. Specifically, in the CS framework, the representation of a signal *f*, via a basis set Ψ, is actually “summarized” by the encoded, compressed version *y* through a measurement/sensing procedure, hence, the term CS. Expanding on this parallelism, we next search for basis and measurement sets in the hippocampus and consider their conformation with the basic constraints (see Section Encoding) of the CS theory.

### Entorhinal cortex

EC comprises the main source of incoming/sensory information for the hippocampus. It is divided into two distinct subregions, the Lateral EC (LEC) and the medial EC (MEC) (Canto et al., 2008). Both DG and CA3 receive excitatory input from LEC and MEC (Figure 4; Witter et al., 2000). Despite their similar architecture, it has been suggested that these two subregions implement different functions (Van Cauter et al., 2013). In particular, there is a consensus that LEC and MEC integrate nonspatial (sensory) and spatial information, respectively (Hargreaves et al., 2005). Based on such findings, it was proposed that the hippocampus receives action (motor) and cue (sensory) information from MEC and LEC subregions, respectively (Lisman, 2007). Motor and sensory information are exploited for the accomplishment of hippocampal functions like spatial orientation (McNaughton et al., 2006), associative learning Gruart et al. (2006), and object recognition (Clarke et al., 2010). It should be noted, however, that motor-related information (such as eyelid position or velocity) is not necessarily encoded by hippocampal neurons Múnera et al. (2001). In this work, the term “motor,” refers primarily to the information related to the space navigation task as reflected by, e.g., the activity of grid cells in MEC.

The following example highlights the EC features that conform to the CS theory. Assume an environment with fixed geometric structure. The spatial information carried by MEC would be mainly deterministic while cue changes within this environment signaled by LEC will have a high degree of stochasticity/randomness. For example, consider a person navigating through his/her home. The familiar, fixed geometry of the rooms will correspond to an already formed map of space (and respective place cell activity). This deterministic spatial information would be signaled to the hippocampus via MEC. Slight changes in the environment, like object displacements and lighting variation create stochasticity in the sensory signals which is assumed to be carried by LEC inputs. Thus, the hippocampus is able to process and store information that depends not only on deterministic facts about the position or state of the subject (MEC), but also on environmental conditions that are highly stochastic/random (LEC). Similarly, the CS theory deals with situations where random sampling of signals (environment) can lead to perfect reconstruction (reconstruction of memories that are associated with the environment), provided that the initial signal has a deterministic, sparse representation (status) in a certain basis set (Ψ ≡ map of the environment). Subsequently, how is sampling/sensing expressed in the hippocampal formation and what is the basis set that defines the position/state of a subject in accordance with motor information?

#### LEC

As previously mentioned, LEC is evidenced to project sensory information to hippocampus (Gnatkovsky et al., 2004). Thus, LEC can be considered to play the role of matrix Φ, as presented in CS. The activity of LEC can be considered as the measurement/sensing media of the environment which is delivered to DG and CA3 via the perforant path (Figure 4). In accordance with the sensing role of LEC, it has been shown that lesions in LEC-hippocampal inputs, thus, ineffective sensing of the environment, led to malfunctions on novelty detection (Myhrer, 1988). In parallel, under the CS framework, ineffective sampling does not capture the whole information spectrum of the signal leading to dysfunctional reconstruction. Thus, LEC can be considered to provide hippocampus with a sampled (sensed) version of the environment. However, the exact mechanisms under which information in LEC represents a sampled version of the environment and what is the role of randomness in this sampling process, remains an intriguing question. All in all, sensing is random in terms of the acquisition of random cues of the environment, including changes that occur to a specified, already learned space and its deterministic structural properties; these cues are reflected in the activity of LEC (firing rates and population coding). However, randomness is just a sufficient condition for effective measurement matrices in CS theory and not a necessary one. The relevant necessity on that, according to the CS framework, is that the measurement matrix and the sparsity basis are incoherent. In this case, it is possible to construct a matrix Φ (LEC activity) that is incoherent to a known basis set Ψ (MEC activity, e.g., grid cells). Whether LEC activity is inherently incoherent to MEC activity remains unclear and is a subject worth further investigation.

#### MEC

Except for the sensing orienting inputs to hippocampus, EC provides information for place-modulated activation of neuronal patterns. Thus, activation patterns, e.g., in DG or CA3, depend on the precise place of the subject in reference with the environment. This position-dependent encoding in hippocampus has its origins primarily on the grid cells lying in MEC (Hafting et al., 2005). Grid cells represent a type of place cells (O'Keefe, 1976) but with a periodic firing in space instead of place-specific firing. The place field of grid cells forms a triangular array (i.e., grid) that expands throughout the whole environment explored by the subject (e.g., a rodent). Each grid is characterized by the spacing, i.e., the distance between the firing fields, the orientation (slope relative to a reference axis) and the phase (displacement relative to a reference axis origin) (Moser et al., 2008). It has been shown that these three variables of a grid field vary in different ways across the MEC (Hafting et al., 2005) but may, to a large extend, be based on hardwired network mechanisms (Hafting et al., 2005). Hence, the instantaneous activity of the grid cells, and thus, of the MEC, resembles the instantaneous value of a sinusoidal signal at specific time point. Thus, each grid cell can be considered as a component of a basis set that consists of items with various spacings, orientations, and phases, much like a sinusoid can be considered as the component of a Fourier basis set with various frequency components. These features are the main criteria that determine whether a component will take part in the representation of a specified signal. Consequently, the activity of the MEC might change in terms of firing expressions through time, but the features that characterize each grid cell, i.e., each component of the basis set, do not change. Actually it has been proposed that grid fields of different spacing, which can be considered as periodic basis functions, combine linearly to generate place fields in the hippocampus(O'Keefe and Burgess, 2005; Fuhs and Touretzky, 2006; McNaughton et al., 2006; Moser et al., 2008). Based on the above, we suggest that for the hippocampus, MEC activity carries the meaning and the functional concept that Ψ represents in the CS theory.

According to the CS theory, the matrix that is associated with the encoding and the decoding processes is the *A* matrix that combines the properties of Φ and Ψ. It would be more convenient if we could assume the projection of EC to hippocampus as a unifying activity and not as two distinct influences from MEC and LEC, respectively. Indeed, it was recently proposed (Van Strien et al., 2009) that due to the overlapping afferents of EC and the interconnections of MEC and LEC, spatial information in MEC and nonspatial (sensory) information in LEC may be already associated as early as at the stage of EC processing. This association supports the assumption that EC provides hippocampus with the neuronal activity that can be parallelized with the contribution of matrix *A* in the CS framework.

### CA3

The proposed manifestation of matrix *A* by the EC provides insights about the possible role of DG and CA3 in the encoding or decoding processes described in CS. As depicted in Figure 4, CA3 receives two main inputs, one from DG via the mossy fibers (MF) and one directly from EC via the perforant path (PP). Thus far, we assumed that the whole encoding-decoding process according to CS takes place in the EC-DG-CA3 circuit and claimed that the final compressed, encoded information is incorporated in CA3. Thus, activity patterns in CA3 could represent vector *y* as described under the CS framework. The proposed mapping between CS (Figure 1B) and the EC-DG-CA3 circuit is graphically depicted in Figure 4.

A number of studies suggest that the recurrent connectivity of CA3 pyramidal cells enables CA3 to act as an autoassociative network (Treves and Rolls, 1992; Lisman et al., 2005) or a heteroassociative one (Lisman, 1999; Cheng, 2013). Hence, with an appropriate initial activation pattern, recurrent connections within CA3 can perform either a pattern completion task (autoassociation) or a transition to a new, subsequent state of a sequential order of states (heteroassociation). In both cases, an association is formed between the current activation pattern and the initial one. The ability to achieve this association has been suggested to depend on the total amount of information stored in the initial pattern, relative to the total number of neurons (Rolls, 2007). Specifically, it has been shown that in terms of efficient transition from the initial pattern to the associated one, the information stored in each firing pattern, *i*_*p*_, must satisfy the inequality:

(4) $$i_{p}>k\ln\;1/k$$

Where *k* stands for the sparsity of the activation pattern in relation to the population size (Rolls, 2007). This lower boundary for *i*_*p*_ is consistent with the lower boundary of the sample number required for efficient reconstruction (Equation 2) in CS theory. Despite the fact that Equation 2 determines only the number of samples acquired randomly from the signal, it can be implicitly related with *i*_*p*_ as the sparsity measure, *k*, reflects both the size of the neuronal population, *N*, and the firing properties (firing rates *r*) of the active population. Specifically, *k*, is defined as per (Rolls, 2007):

(5) $$k=\sum {\left(\frac{r}{N}\right)}^{2}/\sum \frac{r^{2}}{N}.$$

The requirement for effective association between patterns in (Equation 4) must be imposed by an afferent input to CA3. In the case of CS, *M*, the number of required measurements/samples, is determined by the sparsity of the basis set, *K* (see Equation 2). In hippocampus, it was suggested (Treves and Rolls, 1992) that the requirement in (Equation 4) is satisfied by the mossy fibers' synapses on CA3. Mossy fibers, originating from DG, function as detonators on CA3 and impose both the sparse activation and the appropriate information transfer. The above described consensus indicates that the sparse vector *x* is represented by the activity of the DG. This, completes the representation of the encoding step of CS, i.e., *y* = *Ax*, by interpreting the activity of CA3 (*y*) as a result of the interaction of EC (*A*) with DG (*x*) and CA3. As previously mentioned, the implementation of the equation *y* = *Ax* of CS formulas, can be interpreted in the hippocampal network as the concerted influence of regions EC and DG to the CA3 region.

## *L*_1_ minimization by neural circuits

So far, the interpretation of the hippocampal function in terms of the CS theory concerns the encoding phase depicted in Figure 1B. The decoding process depends on an optimization procedure called *L*_1_ minimization (Equation 3) (Candes and Tao, 2005), whose goal is to approximate vector *x*, a task often called sparse approximation. Thus, the next question is whether neural circuits are capable of implementing *L*_1_ minimization and where does this process take place in the hippocampus?

### Locally Competitive Algorithms (LCA)

The first attempt for a neurally plausible *L*_1_ minimization algorithm was made by competitive neural circuit architectures (Rozell et al., 2008). The main principles that govern the proposed architecture are the local competition between neurons in a population and the thresholding that leads to the activation of a subset of neurons that exceed a specified threshold. Thus, the proposed Locally Competitive Algorithms (LCA) facilitate sparse approximation through neuronal populations that continually compete within a restricted area using lateral, mostly one-way, inhibition. Thresholding of the firing rate of the aforementioned population leads to sparsely active neurons that represent the coefficients (*x*) that describe an input signal using an overcomplete dictionary (*M* < *N*). It was shown both theoretically (Balavoine et al., 2012) and by implementation using integrate and fire neurons (Shapero et al., 2013) that LCA corresponds to a robust sparse approximation problem that accounts not only for the minimization of the error depicted in (Equation 3) but also for the sparsity of the solution.

In sum, LCA provide a neurally-based methodology for implementing an *L*_1_ minimization process that is consistent with the CS framework. The question that remains is whether such an algorithm can be implemented in the hippocampus. We propose that DG is a suitable candidate region where this function can be performed.

### Dentate gyrus

Sparse representations are the common way of exhibiting memory oriented activity in DG. Cellular studies have indicated that sparse populations of granule cells, the main encoding cells in DG, are concisely activated, not exceeding 2–4% of the total population (Schmidt et al., 2012). This sparsity enhances the ability of DG to perform one of its most valuable functions during memory formation: pattern separation. Pattern separation guarantees that two separate inputs from EC, even slightly different from each other, are coded by two separate activation patterns in CA3 (Bakker et al., 2008). In CS terms, pattern separation refers to the fact that measurements, *y*_1_ and *y*_2_ of different signals, *f*_1_ and *f*_2_ are due to the different representations, *x*_1_ and *x*_2_, of these signals according to the basis set, Ψ. In the hippocampus, the DG is capable of retrieving the unique sparse representation (*x*) of the cortical input (*f*), according to the grid cells basis (Ψ) of MEC and the sensing information of the environment (LEC ≡ Φ). As these unique representations are uncovered via the *L*_1_ minimization process in the CS framework, the possibility of DG performing such a task is investigated next.

Given that LCA algorithms constitute a proved paradigm of an artificial neural circuit that performs *L*_1_ minimization (Balavoine et al., 2012; Shapero et al., 2013), realization of their key properties by the DG circuitry would reveal the possibility that DG networks express *L*_1_ functions. Interestingly, sparse coding via “competitive learning” in a lateral inhibition framework in DG has already been documented (Ewell and Jones, 2010). Specifically, granule cells excite different kinds of DG interneurons, which in turn inhibit other granule cells of the same cluster (e.g., neighboring cells) (Myers and Scharfman, 2009), enabling the implementation of a locally competitive learning task, one of the main principles of LCA algorithms. The specific role of each kind of interneurons in the *L*_1_ minimization process however remains an open, intriguing question.

Moreover, it is possible that active DG neurons are the result of a thresholding process like the one imposed within LCA networks. More precisely, it has been conjectured (De Almeida et al., 2009) that gamma cycle, a fundamental frequency component in DG and the hippocampus in general, plays a crucial role in the firing task. The critical step is the “search” for the most excitable neurons which become active as inhibition decays during the gamma cycle, followed by those who are less excited (i.e., less tuned to the input). The ordered firing also enhances the sparsity of the outcome, as cells that fire first impose inhibition to other cells and force them to remain silent. This theory proposed by De Almeida et al. (2009), expresses a situation where thresholding is not performed with a fixed predefined threshold but allows for a dynamical tuning of it, so as to enable only the most excited percentage of the population to fire. This process of conditionally tuned thresholding is also adopted by many other algorithms for *L*_1_ minimization (Maleki and Donoho, 2010), revealing the potential *L*_1_ functionality of biological neural circuits. All in all, the abovementioned evidence point to DG as an ideal testbed for *L*_1_-minimization algorithm implementation in the hippocampus.

## Memory storage and retrieval: a model

The association of the CS theory with the main properties of the three different subregions, EC, DG, and CA3, unravels the hypothesized, component-wise manifestation of CS in the hippocampus. Nevertheless, an abstract model of the role of each region in the encoding and decoding phases, during memory formation, needs to be more comprehensively described. In the following paragraphs we describe a novel model according to which encoding as per CS (Figure 1B) constitutes the retrieval phase in hippocampus and decoding corresponds to memory storage. Thus, given an input from EC (matrix *A*) and an active projection from DG to CA3 (vector *x*), an already learned memory (vector *y*), is represented by the produced activity in CA3 (i.e., *y* = *Ax*). If the EC input carries new information (i.e., new memories) error terms (depicted as noise in Figure 1B, also see below) are produced and DG performs *L*_1_ minimization in order to provide a new vector *x* (appropriate active population in DG) that forms/stores (through mossy fibers) new memories in CA3. These storage and retrieval phases are further explained below. In general, the proposed model intends to show that already established functional properties of the hippocampal subregions coincide with the unifying theory of CS and, specifically, that CS provides the theoretical framework to interpret the concerted interaction of different functions (pattern separation, pattern completion etc.) to form hippocampal memories.

### Storage

The formation of memory engrams as these are represented by active population patterns and connectivity relations in DG and CA3, is based on the fundamental principle that different memories must be stored in different patterns. As previously mentioned, pattern separation is a crucial function of DG and is mostly based on its sparse activity which also provides orthogonal representations in CA3 (Treves et al., 2008). Thus, rarefying the active population in DG according to the given information (from EC), represents a good approach for effective orthogonalization of memory engrams.

In CS, this is accomplished by revealing the sparsest representation, *x*, of the signal, *f*, in basis Ψ, according to the information provided by vector *y* (measurement). Under the CS framework, the sparse representation of signal *f* in Ψ is a prerequisite. However, due to the abstract status of the *f* entity in cortex, which is intended to be stored/encoded in the hippocampus, sparsity is not predefined but can be imposed by the hippocampal circuitry, in order to exploit benefits that have to do with pattern separation issues but also with energy consumption. This can be thought of as an ill-posed mathematical problem. For instance, someone is thinking of two integers whose average is 5; what are those numbers? Of course there are multiple answers to this question but [10, 0] is the sparsest one. In case that these numbers are firing rates of neurons, only one out of two neurons is needed to fire to meet the context of the algebraic query (average 5). The above-described rationale facilitates the need for a process that imposes a sparse representation in DG, with respect to the basis' information derived from the EC. The *L*_1_ minimization process is proposed here as a memory storage mechanism that sparsifies the DG activity subject to the context (information measured from the environment). As discussed above (see Section Dentate Gyrus), the implementation of such a process by the DG circuitry is plausible. In sum, we propose that the hippocampal circuitry uses an *L*_1_ minimization process to estimate a sparse representation of the initial cortical state. This process takes place in the DG taking into account the compressed version of signal *f*, i.e., vector *y*, which is expressed in CA3. The sparsification of the initial signal provides not only with the computational benefit of processing compressed versions of it during its pass from hippocampus, but also enables the “reconstruction” of the cortical representation when the hippocampal outcome is fed back to the EC (see Section The role of CA1).

**Figure 5:**
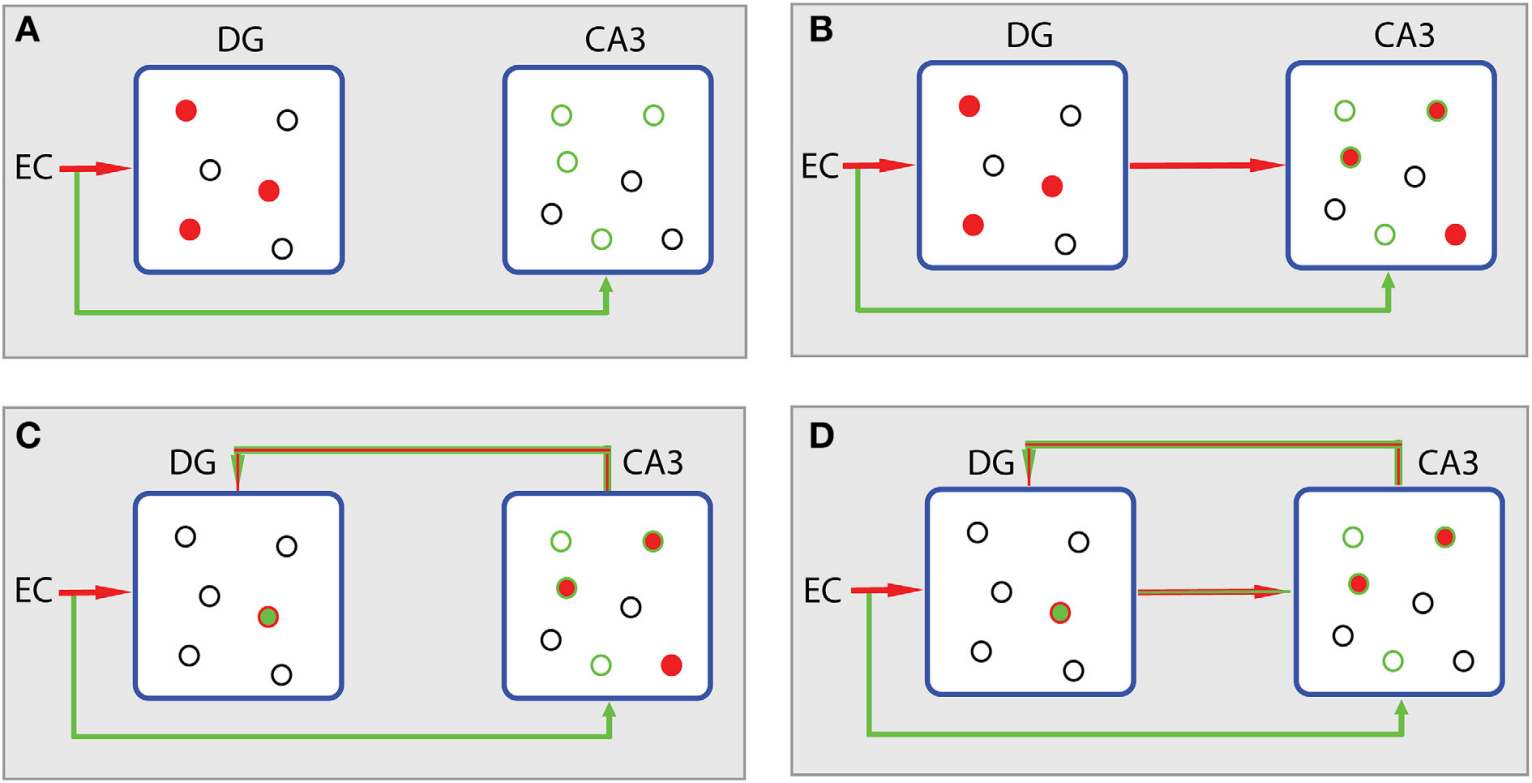
**Processing scheme in hippocampus according to the CS theory. (A)** The EC input causes the firing of a population of granule cells in DG (filled red cycles) and a subthreshold depolarization of a subset of CA3 pyramidal cells (empty green cycles). Source of excitation and corresponding activity are depicted with the same colors. Excited cells are depicted by filled cycles. Colored empty cycles represent near-thershold cells, which are depolarized but not active. Black cycles represent cells at rest. **(B)** In CA3, excited cells, the ones excited after the subthreshold depolarization and the ones directly excited by mossy fibers (filled red cycles in CA3), constitute the joint contribution of EC (matrix *A*) and DG (*x*′) to CA3 activation (*Ax*′). The noisy subset *y*′ of neurons is expressed by the subthreshold depolarized cells (green cycles in CA3). **(C)** The error term produced in CA3 (‖*Ax*′ ‒ *y*′‖_*L*_2__) is fed back to DG in order to participate in the *L*_1_ minimization algorithm taking place in DG. DG's activity leads to the sparsest population (min ‖ *x*′‖_*L*_1__) that meets the demands of the contextual information (green empty cycles in CA3) from which current activity of CA3 (filled cycles) diverges. **(D)** The algorithm evolves with the incorporation of the sparser projection from DG to CA3 causing a new *Ax*′ activity in CA3 (filled cycles).

Figure 5 summarizes the different storage steps according to the *L*_1_ minimization concept. The reasoning adopted here is actually an enhanced version of the context-based model of episodic memory formation proposed by John Lisman (Lisman, 1999). Assume that the EC input to DG and CA3 via the perforant path causes the firing of a population of granule cells and a subthreshold depolarization of a subset of CA3 pyramidal cells (Figure 5A). Information conveyed to CA3 can be differentiated from that sent to DG. It is assumed to deliver contextual content that is not expressed by the explicit firing of the CA3 cells, but by the positively biased potential of the forthcoming excitation through the mossy fiber afferents (Lisman, 1999). Then, apart from the excitation of the positively biased pyramidal cells in CA3 (Figure 5B, green cycles), other CA3 cells may also fire due to the strong mossy fiber connections (Henze et al., 2002). These cells, the ones excited after the subthreshold depolarization and the directly excited ones, constitute the joint contribution of EC (matrix *A*) and DG (*x*′, where *x*′ is just an estimation of signal *x*) to CA3 activation. The noisy subset *y*′ of neurons (conceptually described in Figure 1B) is expressed by the subthreshold depolarized cells (green cycles, Figure 5A). Thus, the divergence of the joint EC-DG (*Ax*′) effect on CA3 from the subthreshold activated CA3 population (*y*′) stands for the error minimization term described by the second part of equation (Equation 3), i.e., ‖*Ax*′ − *y*′‖_*L*_2__ ≤ ε.

If we want to store a memory in CA3 that is related to the context, presented by the biased CA3 population, with as sparse representation in DG as possible, these two regions must be reciprocally connected. Indeed, except for the direct projection of DG to CA3 there is also a backprojection path from CA3 to DG (Lisman et al., 2005; Scharfman, 2007; Figure 4). The role of this backprojection on pattern separation in the DG was previously investigated (Myers and Scharfman, 2011) revealing its contribution to sparsity through inhibition. Based on this evidence, we propose that the error term (noise) produced in CA3 is fed back to DG in order to participate in the *L*_1_ algorithm, i.e., the effort to find the sparsest population in DG (*min* ‖*x*′‖_*L*_1__, see Equation 3) that meets the demands of the contextual information from which the current activity of CA3 diverges (Figure 5C); in other words, to perform the minimization task described by Equation 3. Then, the algorithm evolves and the next step incorporates the sparser projection from DG to CA3 (Figure 5D) causing a new *Ax*′ joint effect.

It should be stressed out that there is no need for the final active population to cover the entire *y*′ population (the contextual content of information described by *y*′ neurons in CA3). In other words, the same contextual information may take part in different potential memory engrams. In fact, vector *y* is considered as the final active population of CA3 (Figure 1B) and, hence, the whole storage process through the *L*_1_ algorithm, also performs a “denoising” action which is critical for the decoding/retrieval process.

All in all, the estimation of *x* through the aforementioned process is vital for the storage/encoding phase. Actually, the final, refined encoding of the cortex state *f* is imposed to CA3 by its sparse representation, *x*, instead of the whole redundant cortex representation. This reveals once again the need for sparsification of the initial cortical state *f*. Upon retrieval (decoding) of a specific memory, the sparsified version of the initial information entity(*f*), namely signal *x*, is sufficient to recall the final activity state of CA3, namely signal *y* (see also subsequent “Retrieval” section). Usage of a sparsified version of signal *f*, i.e., *x*, instead of the whole representation, greatly reduces the computational effort of hippocampus, especially when sequences of heteroassociated memories are stored/processed.

### Retrieval

The process of memory retrieval according to the proposed model is basically a static *L*_1_ minimization. In other words, the error term (noise) produced in CA3 causes no change in DG activity and consequently CA3's activity remains approximately the same. As a result, the joint contribution from EC and DG (*Ax*′) produces the, already stored, pattern *y*. In an episodic memory retrieval case, multiple, sequential *y* populations must be retrieved, corresponding to different episodes of the memory. The heteroassociation between the different *y* populations and the autoassociation (pattern completion) can be accomplished by the reciprocal connection between DG and CA3 (Lisman et al., 2005) whereas DG can contribute, by the process previously discussed (*L*_1_ algorithm), to the correction of probable errors. For instance, if the needed heteroassociation is *y*_1_ → *y*_2_ and there is an error term causing *y*′_1_ instead of *y*_1_, this error would be propagated and enhanced, to the next pattern causing *y*″_2_ (number of primes stands for the level of noise). Nevertheless, this can be avoided by the reciprocal information exchange between DG and CA3, which is possible to be performed a few times before the heteroassociation step (Lisman, 1999). In sum, the retrieval phase of the proposed model is assigned to the encoding step of the CS framework, where *A* and *x* explicitly produce *y* (without additional *L*_1_ processing), namely the compressed version of the initial “signal” *f*.

### The role of CA1

In a previous paragraph it was conjectured that the hippocampus exploits the benefits of CS by creating a sparse representation of the initial cortical information, according to the basis set and sensory cues provided by the EC. Based on this, memories are encoded/stored in CA3 in condensed neuronal populations, allowing for manipulation and heteroassociation of compressed embodiments of the initial information formed in the cortex. Thus, a compressed version of the more complex representation in the cortex is used during processing in the hippocampus. This is also one of the main contributions of CS in information theory: enabling the efficient processing of a measured, compressed version of the initial signal without the need for its full representation (Davenport et al., 2010). It can be conjectured that hippocampus has evolved to exploit this particular benefit instead of processing widespread cortical information of episodic memory engrams.

Moreover, the outcome of hippocampal tasks is fed back to the EC probably for further processing and/or for updating the EC status (match/mismatch computation) (Lisman, 1999). According to the model proposed here, the sparsity of the cortical signal *f* is not predefined but rather imposed by the hippocampus in accordance with the information received from EC; thus, the basis sets in EC must be updated accordingly. In agreement, it was recently shown that grid cell formation in MEC is affected by the hippocampal feedback (Bonnevie et al., 2013).

Importantly, we do not claim that the signal reconstruction phase described in CS is faithfully reproduced in the EC-GD-CA3 circuit. What we propose is that compressed engrams formed in the hippocampus are transformed to the redundant forms of cortical signals through a fanning out process using the same *alphabet* (dictionary set); the exact reverse process takes place during the EC to DG to CA3 information transfer. In other words, incoming cortical signals are compressed, processed and then transformed into a conceivable representation that can be read out by the cortex (Lisman, 1999). It should be stressed that the possibility of signal reconstruction from random projections (measures) to the initial form of information (see Section CS Basics) can be realized under the assumptions of the CS theory and the sparsity constraint.

The hippocampal region that mediates the abovementioned feedback from hippocampus to EC is the CA1 (Figure 4). This region receives input directly from CA3 and EC (layer III) and projects back to different layers (V and VI) of the EC (Witter et al., 2000). Thus, by incorporating the information from CA3 (*y*) and EC (Φ and Ψ from LEC and MEC, respectively, albeit via a different route), we propose that the CA1 area can convert the compressed information to the same form as the initial information that was processed. This closes the information loop which incorporates the compression and decompression of messages while passing through hippocampus with a profound benefit: the low computational cost yet effective information processing.

## Conclusions and future considerations

This work provides extensive evidence in support of a new theoretical framework that explains hippocampal processing. Unlike previous theories that lacked a unifying, mathematically formulated view of inter- and intra-regional relationships, we propose that the EC-DG-CA3-CA1 circuit operates under the conditions and with the advantages of compressed sensing, a recent breakthrough in signal processing.

The CS perspective provides revolutionary insights regarding the interpretation of hippocampal function. Specifically, this paper provides extensive evidence that redundant cortical signals are compressed within the hippocampus in order to be manipulated faster and more efficiently before sent back to the cortex. Encoding and decoding phases are actually the two sides of the same coin, which is essentially the transform from a coarse/redundant to a condensed version of information. Heteroassociation of these compressed information packages underlines a key hippocampal function, namely the ability to form episodic memories.

In addition to a novel view of hippocampal processing, this perspective has a number of contributions that can lead to theoretical and experimental investigations needed to corroborate the CS theory. These include (a) the predicted *L*_1_ minimization realized by DG and its critical role in memory storage; (b) the prediction that different inhibitory cells in the DG contribute to the realization of this process; (c) the prediction that the EC processing is characterized by the RIP property; (d) the predicted association between LEC and MEC signals dictated by the incoherency property, and (e) the predicted updating of EC basis sets achieved via the CA1 backprojection. In the next paragraphs we discuss the importance of these contributions and suggest ways for their further investigation.

The adoption of the CS framework as a representative theory of hippocampal function provides a verifiable ground truth against which the contributions of various circuits in the hippocampus can be tested. For instance, the proposed *L*_1_ minimization function of DG opens new avenues for dissecting the role of different cell types (mossy cells, hillar cells, etc) in DG processing. Based on this proposition, one can expand the LCA network by adding cell-type specific features of the DG circuitry and investigate their impact on the *L*_1_ minimization task. In addition, it would be crucial to investigate the role of neurogenesis that takes place in DG on the same task, via incorporating for example neurogenesis in the abovementioned models. Specifically, it has been suggested that newly generated granule cells during adulthood modulate local network inhibition (Sahay et al., 2011; Kheirbek et al., 2012), which constitutes one of the fundamental features of the LCA architecture. Experimental studies could be designed to manipulate neurogenesis in animals and assess the effect of these manipulations on the sparsity of DG activity and the storage/retrieval capacity of the hippocampus as foreseen by CS.

The RIP property, a prerequisite for the *A* matrix that is linked with the activity of EC, paves the way for more efficient calculation of memory-related parameters. A recent study used the RIP mathematical formalization (Charles et al., 2014) to show that memory capacity of randomly connected recurrent networks (like the ones in CA3) receiving inputs that are approximately sparse in some basis, can scale superlinearly with the number of neurons. Moreover, under certain conditions, memory capacity was found to largely exceed network size. While RIP has yet to be proved for the EC projection to the hippocampus, evolutionary aspects support the plausibility of such an assumption. In essence, the RIP property ensures that two memories represented by two distinct activation patterns in DG map onto two separable representations in the CA3. In other words, the EC activity, due to the RIP property, enhances the pattern separation task. This can be tested experimentally using optogenetic stimulation of the EC in animals trained to learn two distinct memories and looking at the overlap of the cellular populations that capture each memory with plasticity markers (Ramirez et al., 2013). Computational models could investigate this prediction by looking at how pattern separation is affected by manipulations of the RIP property in EC inputs.

Moreover, incoherency, as interpreted in a previous section, implies an association between LEC and MEC. Conceptually, information propagated to the hippocampus from MEC can be regarded as incoherent with the one projected by LEC due to the geometric (grid cells) and non-geometric (contextual) information represented by each subregion, respectively. Furthermore, since contextual information was shown to affect place field formation (Anderson and Jeffery, 2003), it would be intriguing to investigate if the incoherency between LEC and MEC is not only conceptual but also activity oriented, i.e., in terms of firing patterns and correlation of populations' activity. For example, recordings from LEC and MEC during the performance of a memory task could be used to test whether the activity of the two regions is incoherent. The incoherency, from the mathematical perspective, may provide valuable insights on this investigation.

Furthermore, we propose that the CA1 backprojection ensures that the EC basis components, used for the storage of a new memory, are selected again (see Section Retrieval), without learning (i.e., no plasticity in the DG), upon subsequent presentation of this memory (retrieval). Experiments could be designed to test this hypothesis by lessioning the CA1 backprojection and measuring the levels of plasticity in DG along with the CA3-DG backprojection (error signaling).

Besides the benefits that may be gained from the neuroscience point of view, engineering aspects could also be affected by the CS perspective of hippocampal function. For instance, new *L*_1_ minimization algorithms can emerge from the DG circuitry interpretation. Moreover, robot navigation systems, can be constructed based on the hippocampal functioning. Systems that exhibit similar architecture with the hippocampus have been suggested (Verschure et al., 2003) and better interpretation of hippocampal functioning will lead to further enhancement of such systems.

To conclude, the CS framework seems to align with various aspects of hippocampal function. In addition, it provides analytical tools to breakdown and compartmentalize its contribution to neural information processing. A complete understanding of the hypothesized CS manifestation by the hippocampus, demands both experimental and theoretical work and is likely to lead to reexamination of various aspects of information flow in the brain.

### Conflict of interest statement

The authors declare that the research was conducted in the absence of any commercial or financial relationships that could be construed as a potential conflict of interest.
